# Malaria: Knowledge and prevention practices among school adolescents in a coastal community in Calabar, Nigeria

**DOI:** 10.4102/phcfm.v2i1.103

**Published:** 2010-04-16

**Authors:** Ndifreke E. Udonwa, Abraham N. Gyuse, Aniekan J. Etokidem

**Affiliations:** 1 Department of Family Medicine, University of Calabar Teaching Hospital, Nigeria; 2 Department of Community Medicine, University of Calabar Teaching Hospital, Nigeria

**Keywords:** malaria, prevention, adolescents, coastal community, Nigeria

## Abstract

**Background:**

Malaria prevention and treatment constitute an unbearable economic burden to most African countries, especially south of the Sahara, where about 500 million cases occur annually. The problem of malaria among adolescents has largely been overshadowed by the huge burden of the disease among young children. Attention to malaria among adolescents has also been diverted by the huge burden of HIV/AIDS among adolescents. Some surveys reveal a lack of knowledge and many misconceptions about the transmission and treatment of malaria, which could adversely affect malaria control measures and antimalarial therapy. Such a knowledge gap could have an adverse effect on school children, who could be used as change agents and as role models for their siblings and peers in the malaria control strategy.

**Objectives:**

To determine the malaria prevention practices of school adolescents in the coastal community of Calabar, Nigeria.

**Method:**

This was a cross-sectional survey involving secondary schools in southern Calabar. Four hundred adolescents were randomly selected from the 4565 learners in 5 out of 17 secondary schools in southern Calabar, Cross River State, Nigeria. A self-administered, semi-structured questionnaire was administered to the respondents.

**Results:**

Most respondents (77.5%) were aware that the vector transmits the malaria parasite through biting. Fewer respondents would prevent malaria attacks by clearing the vegetation in the peri-domestic environment (13.5%), filling up potholes (16.9%), opening up drainage (11%), using insecticide-treated nets (25.7%) or using antimalarial drugs (11.2%). Less than one-tenth (8%) would use various other methods such as not accepting unscreened blood, while only 11% obtained the information from their teachers.

**Conclusion:**

The study identified knowledge gaps among school children. There is a need to empower teachers with information about the cause of malaria and prevention strategies.

## INTRODUCTION

More than 20% of humanity is affected by malaria.^1^ The human and economic costs associated with declining quality of life, consultations, treatments, hospitalisation and other events related to malaria are enormous and often lead to low productivity and lost incomes.^2^ In sub-Saharan Africa, where 90% of the world's malaria occurs, about 500 million cases are recorded annually with hundreds of thousands of child deaths. In Nigeria, like in many west-African countries, malaria is a major cause of morbidity and mortality. It is estimated that over 50% of Nigerians suffer at least one bout of malaria every year.^3^

Among school adolescents, malaria is responsible for school absenteeism, poor performance in school, examination failures, school dropouts and even death. The problem of malaria among adolescents has largely been overshadowed by the huge burden of HIV/AIDS among this younger age group.^4^ The younger age group has been identified as bearing half of the burden of HIV worldwide.^5^ As much as 60% of school children's learning may be impaired by malaria.^6, 7, 8, 9, 10^

Experiences with malaria have shown that prevention is better and cheaper than cure; however, the practice of malaria preventive measures has been related to the knowledge and belief of people.^11^ Malaria-related knowledge, attitudes and practices have been examined in many rural and partly urban multi-ethnic populations in Africa.^12, 13, 14, 15^ Within Nigeria, surveys of residents of the Atlantic coast revealed a lack of knowledge and many misconceptions about the transmission and treatment of malaria, which could adversely affect malaria control measures and antimalarial therapy.^16^

The 1998 Roll Back Malaria (RBM) initiative, launched in Geneva by the United Nations Children's Fund (UNICEF), the United Nations Development Programme (UNDP), the World Bank and the World Health Organization (WHO), is a people-oriented programme that emphasises community participation. School children could contribute immensely to its success. Malaria-intervention goals in endemic areas should be to prevent mortality and reduce morbidity, as well as associated socio-economic losses. This requires the progressive creation of capacities for assessing the local malaria situation and selecting appropriate control measures.^2^ Correct knowledge of a health problem, when combined with the right attitude, can lead to healthy behaviour and practice.^17^ Being the most educated in most rural communities, school adolescents’ knowledge, attitude and practice regarding a health problem can easily influence those of their peers, parents and other members of their communities.

School adolescents, therefore, constitute a formidable community entry point for the control of malaria under the people-oriented malaria control strategy, the RBM programme. A critical mass of knowledgeable school adolescents, with the right attitude towards the RBM programme, could act as a catalyst for community involvement in rolling back malaria. This is critical for the right practice of the RBM programme as well as programme sustainability. Studies of malaria-related knowledge, attitudes and practices, have been widely used in research on malaria and have proved useful tools in shaping policy formulations as well as guiding programme implementation.^11, 12, 16^

In Cross River State in south-eastern Nigeria, malaria is such a significant problem among school children that the state government decided to involve them fully in the implementation of the RBM programme.^18^ This is because school children are known to easily imbibe and implement new knowledge^18^ and can, therefore easily act as change agents and as role models for their siblings and peers.

This study thus sought to investigate the malaria knowledge and prevention practices among school adolescents in a coastal community in Calabar, Cross River State, Nigeria.

## MATERIALS AND METHOD

This was a cross-sectional, descriptive study of malaria knowledge and prevention practices among school adolescents in a coastal community in Calabar, Cross River State, Nigeria. The study area has an estimated population of 191 630.^19^ It is bordered in the west by the Calabar River, in the south by swamps and creeks, in the east by the Great Kwa River and in the north by Calabar Municipality. The area lies between 8°8’ and 8°22’ N and 4°54’ and 4°58’ E and is located within the rainforest belt. The area, therefore, experiences a rainy season from March to October with its peak in July and a dry season from November to February every year. This is probably why the prevalence of malaria is so high in the area.

A multi-stage sampling method was used to select schools and to select respondents from these schools. Five secondary schools were randomly selected from all 17 government and privately owned secondary schools in the local government area (LGA), with a learner population of 12 000. Information about the schools was obtained from the State Ministry of Education and the local government education authority. Five secondary schools were selected, based on their status as a single sex school or a mixed school. The schools were grouped into boys-only schools, girls-only schools and co-educational schools. For this study, two schools were selected randomly from each of the two former categories, while three were selected from the latter.

The five schools selected were predominantly day schools (two of the schools provide accommodation for less than 25% of the learners). Three of the schools (co-educational) were government schools. One girls-only and one boy-only school were private schools.

Four hundred learners were selected randomly out of the 4585 learners who attended these five secondary schools to participate in the study. Since the population of each of these five schools was different, a proportionately representative sample was randomly selected from each of them to reflect their numerical strength.

Permission was obtained from the principals of the schools before embarking on data collection. Five learners were drawn by a systematic random sampling method from each class from JSS1 to SS3 (90 classes) in each of the schools. Informed consent was obtained from the selected learners. Data collection was carried out between October and November of 2007 using a semi-structured questionnaire. The questionnaire included socio-demographic data on the learners and knowledge of malaria and prevention practices.

Data obtained from the study were analysed using the statistical software Epi-Info 2002, CDC, Atlanta, USA.

## RESULTS

All 400 administered questionnaires were returned, giving a response rate of 100%. Of the respondents, 228 (57%) were male while 172 (43%) were female. The age of the respondents ranged from 13 to 19 years, while the mean age was 14.7 (s.d. ±4.2).

Only nine (2.3%) respondents gave an acceptable definition of malaria as an infective disease caused by parasites that are transmitted through the bite of an infected mosquito. The most commonly mentioned local name for malaria was *utoe enyin* (‘yellow eyes’). About half of the respondents knew the plasmodium falciparum, the causative agent, but a similar fraction did not know the vector, the female *Anopheles* mosquito. This is shown in Table 1.

**TABLE 1:** Knowledge of malaria causation among secondary school adolescents in southern Calabar

	Causative agent	Malaria vector	Mode of transmission
		
	*n* (%)	*n* (%)	*n* (%)
Correct knowledge	212 (53)	167 (41.7)	310 (77.4)
Incorrect knowledge	188 (47)	233 (58.3)	77 (19.3)
Non-response	0 (0)	0 (0)	13 (3.3)

**Total**	**400 (100)**	**400 (100)**	**400 (100)**

**FIGURE 1:**
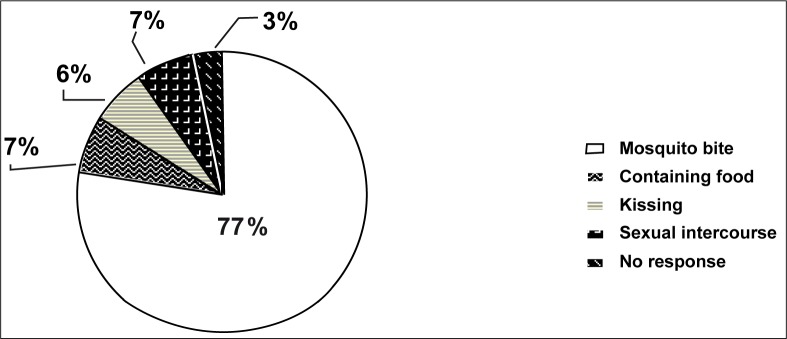
Knowledge of mode of transmission of malaria among secondary school adolescents in southern Calabar

Concerning the mode of transmission of malaria, 310 (77%) of the respondents were aware that the vector transmits the parasite by biting, while 77 (20%) gave other modes of transmission that include swallowing the vector in contaminated food (7%), kissing (6%) and sexual intercourse (7%). Thirteen (3%) of the respondents did not give any response (see Figure 1).

**FIGURE 2:**
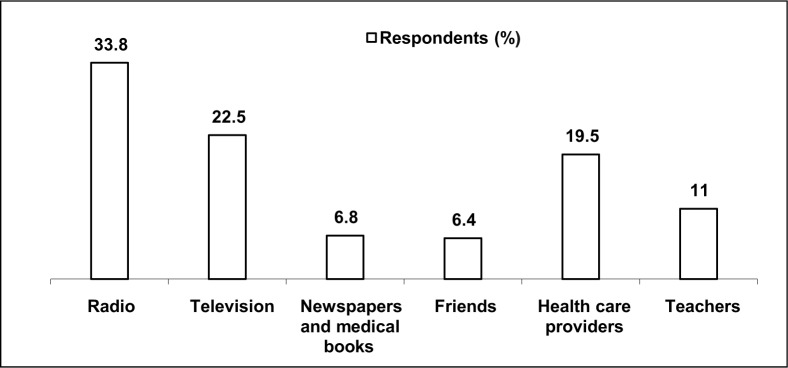
Sources of information about malaria prevention among secondary school adolescents in southern Calabar

Respondents differed greatly on the main source of their knowledge of malaria prevention. In total, 135 (33.8%) had heard about it over the radio, 90 (22.5%) through the television, 27 (6.8%) from newspapers and medical books, 25 (6.4%) from friends and 78 (19.5%) from health care providers such as nurses and doctors, while 45 (11%) had heard about malaria from their teachers (see Figure 2).

In total, 282 (70.5%) of the respondents were aware that insecticide-treated nets (ITNs) kill mosquitoes, while 90 (22.5%) responded that ITNs are used to treat insects. Nineteen (4.75%) respondents indicated that ITNs are used to catch fish, while 9 (2.25%) responded that they are used to trap rats.

Among the respondents, 296 (74%) were aware of a place in their locality where ITNs are sold, while 104 (26%) were not aware of such a place or did not respond. This is shown in Table 2. Out of the 296 respondents that knew where ITNs are sold, 75 (18.8%) mentioned chemists as the place where ITNs are sold in their locality, 179 (44.8%) mentioned hospitals/clinics, while 42 (10.5%) mentioned supermarkets.

**TABLE 2:** Knowledge of places where ITNs are sold among secondary school adolescents in southern Calabar

Place of sale of ITN	Number of respondents	Percentage
Chemist	75	18.8
Hospital/Clinic	179	44.8
Market	42	10.5
No response	104	26

**Total**	**400**	**100**

Concerning the main method of preventing malaria attacks, 54 (13.5%) of the respondents would clear the vegetation in the peri-domestic environment, 67 (16.9%) would fill potholes, 44 (11%) would open up drainage, 103 (25.7%) would use ITNs, 45 (11.2%) would use antimalarial drugs and 32 (8%) would use various other methods, such as not accepting unscreened blood (see Table 3).

**TABLE 3:** Malaria-prevention methods among secondary school adolescents in southern Calabar

Method	Number of respondents	Percentage
Clearing vegetation	54	13.5
Filling potholes	67	16.9
Opening drainage	44	11
Using ITNs	103	25.7
Using insecticide spray	45	11.2
Using anti-malarial drugs	45	11.2
Other	32	8
No response	10	2.5

**Total**	**400**	**100**

**FIGURE 3:**
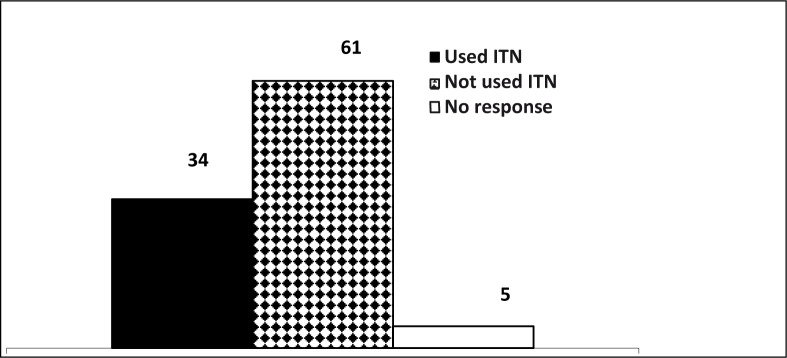
Use of ITNs among secondary school adolescents in southern Calabar

Among those who responded to the use of an ITN as a preventive measure, 34 (33%) had ever used an ITN themselves, while 61 (59%) had never used it (Figure 3).

**FIGURE 4:**
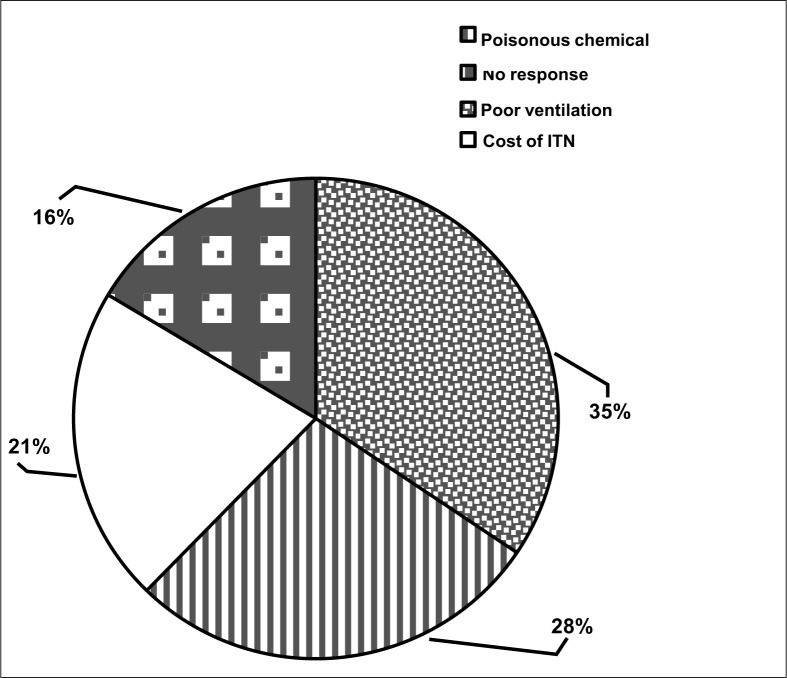
Reasons for refusal to use an ITN

Twenty-one (35%) of the learners refused to use an ITN for fear of death while inside the net due to poor ventilation, while 17 (28%) gave the use of a poisonous chemical for treatment of the net as their reason for refusal to use an ITN (Figure 4). Thirteen (21%) gave high cost of the net as reason for refusal to use it.

## DISCUSSION

This study has shown that there are gaps in knowledge of malaria aetiology. About one-quarter of the respondents gave the local name for malaria, *utoe enyin*, which in Efik (the most predominant local language in the study area) literally means yellowness of the eyes. Yellowness of the eyes is one of the symptoms of severe malaria, but is an early sign and symptom of several other infectious diseases, and it therefore cannot be said to be absolutely indicative of malaria. Out of those who were aware that malaria is caused by the malaria parasite, only about one-third knew that it is only this parasite that can cause malaria, while the majority of the respondents wrongly answered that other agents equally cause malaria, in addition to the malaria parasite. The respondents who did not know that malaria is caused by the malaria parasite were of the opinion that the disease is caused by viruses and bacteria (staphylococcus). This is a serious knowledge gap and is similar to findings among residents of the Atlantic coast in Lagos, which also revealed a lack of, or poor, knowledge of the transmission of malaria.^16^

In a fairly literate population such as the one used for this study, the finding that only nine (2.25%) respondents were able to give a fairly acceptable definition of malaria as an infectious disease caused by the parasitic infection of red blood cells by plasmodium, which is transmitted by the bite of an infected female mosquito, shows a wide knowledge gap that needs to be filled. Only 167 (41.75%) of the respondents were aware that the vector that transmits the malaria parasite is the female *Anopheles* mosquito. This percentage is low compared to the 90% that were aware of this fact in a study in the Pacific coast of Guatemala.^20^ The reason for the absence of this knowledge in the current study is probably because of a low capacity of teachers informed about malaria causation or an inadequate content of health education in the curriculum for the teaching of primary and secondary school children.

This study shows that only 25% of the respondents use ITNs as a preventive measure, with about one-third of them ever having slept under an ITN. This low percentage agrees with that found by Dressa et al. in their study, which found that only 13.0% of their respondents used bed nets as preventive measures against mosquito bites and malaria^14^, a result they found similarly in the Kyela district in south-western Tanzania, where only 17.6% of school children used ITNs.^15^

The poor attitude towards ITN use was due to various reasons. This included death while inside the net because of a lack of air and the belief that the chemical used in treating the net was very poisonous. These perceived disadvantages of ITNs have no scientific basis and can easily be overcome through health education.^21^

A reasonable proportion of the respondents knew that ITNs are sold at chemists and markets. These are sources outside the main health care system and with inherent danger of poor quality control, although they serve many communities in the environment. It is therefore necessary that regulatory mechanisms be put in place to ensure the quality of products reaching the grassroot consumers. The less-than-average sourcing of ITNs from hospital/clinics is an indication of the collapse of the formal health care delivery system, often leading to diversion of these products to the chemists and markets with attendant loss of quality control.

The radio, television and health care providers constituted the main sources of information on malaria prevention. Teachers were a source of information on malaria prevention for only about one-tenth of the adolescents. This very low percentage of representation of teachers is in contrast to the 47.4% found among school children in Tanzania.^15^ This finding suggests the need to empower school teachers with health information so that such information could be passed on to the learners. This may also be indicative of the collapse of the Nigerian educational system, as health education is an integral part of the curriculum at primary and secondary levels.

ITN use is the main mode of malaria prevention given by respondents, followed by the filling of potholes, which ensures the reduction of pools of water where breeding of mosquitoes takes place. The concept of the use of antimalarial drugs such as chemoprophylaxis is common among the respondents, who indicated this as a main method of malaria prevention. Malaria prophylaxis is not recommended for the general population in Nigeria, except for vulnerable groups such as those with sickle cell anaemia and non-exposed expatriates.^22^ Therefore, public health education must be strengthened to re-enforce ITN use and environmental sanitation, while discouraging the inappropriate use of chemoprophylaxis. Close to one-tenth of the adolescents would use various other methods of preventing malaria, including the refusal of unscreened blood, a method that could prevent malaria transmission and one of the methods of HIV/AIDS prevention.

## CONCLUSION

This study showed that a good number of school adolescents in the study area were knowledgeable about malaria transmission. The mass media was a major source of information for adolescents on malaria, while health care providers contributed only modestly. The use of ITNs for malaria prevention was well known by these adolescents and they also knew where they can source it. However, the study identified some gaps in the knowledge of the causative agent of malaria and antimalarial preventive methods at a grassroots level. These gaps must be filled in order to make the adolescents good change agents through involvement in the RBM programme. Even though only a small percentage, the fact that some of the respondents mentioned refusal of unscreened blood indicated that there could be some level of confusion between malaria and HIV/AIDS transmission, although malaria could well be transmitted through blood transfusion. The apparent confusion between malaria and HIV/AIDS transmission must be addressed through intensive public health education among adolescents and, indeed, the entire population.

Owing to the sourcing of ITNs outside the regular health care system, it is necessary that regulatory mechanisms be put in place to ensure the quality of ITNs reaching the grassroot consumers.

There is a need to strengthen the educational system by empowering teachers with information about malaria causation and prevention strategies so that such knowledge could be passed on to learners. The mass media must also continue to perform its function of information dissemination, while the local governments can create television viewing centres at the grassroots level. Primary health care can also strengthen public health information through group counselling and information, education and communication materials.
